# A Text-Book of Practical Therapeutics

**Published:** 1900-12

**Authors:** 


A TEXT-BOOK OF PRACTICAL THERAPEUTICS.—With especial reference to the application of remedial measures to disease and their employment upon a rational basis. By Hobart Amory Hare, M. D., Professor of Therapeutics and Materia Medica in the Jefferson Medical College of Philadelphia. With especial chapters by Drs. G. E. DeSchweinitz, Edward Martin and Barton C. Hirst. New (8th) edition. In one octavo volume of 706 pages, with 37 engravings and 3 colored plates. Cloth, $4.00; leather, $5.00, net. Lea Brothers & Co., Philadelphia and New York.

It is hard to keep up with the editions of Hare, they come so thick and fast—eight in nine years. Hare has become the acknowledged authority on therapeutics. In addition to the work being a standard on remedies, the second half is devoted to applied therapeutics, and is, indeed, a pithy practice of medicine.

